# Network-Centric Interventions to Contain the Syphilis Epidemic in San Francisco

**DOI:** 10.1038/s41598-017-06619-9

**Published:** 2017-07-25

**Authors:** David Juher, Joan Saldaña, Robert Kohn, Kyle Bernstein, Caterina Scoglio

**Affiliations:** 1 Universitat de Girona, Girona, Catalunya Spain; 2 San Francisco Public Health Department, San Francisco, California USA; 3 Centers for Disease Control and Prevention, Atlanta, Georgia USA; 4 Kansas State University, Manhattan, Kansas USA

## Abstract

The number of reported early syphilis cases in San Francisco has increased steadily since 2005. It is not yet clear what factors are responsible for such an increase. A recent analysis of the sexual contact network of men who have sex with men with syphilis in San Francisco has discovered a large connected component, members of which have a significantly higher chance of syphilis and HIV compared to non-member individuals. This study investigates whether it is possible to exploit the existence of the largest connected component to design new notification strategies that can potentially contribute to reducing the number of cases. We develop a model capable of incorporating multiple types of notification strategies and compare the corresponding incidence of syphilis. Through extensive simulations, we show that notifying the community of the infection state of few central nodes appears to be the most effective approach, balancing the cost of notification and the reduction of syphilis incidence. Additionally, among the different measures of centrality, the eigenvector centrality reveals to be the best to reduce the incidence in the long term as long as the number of missing links (non-disclosed contacts) is not very large.

## Introduction

Since 2001, San Francisco has experienced a sustained syphilis epidemic that has been nearly exclusively limited to men who have sex with men (MSM)^1^. The epidemic, which was declining a few years ago, is now experiencing a new resurgence, not only in San Francisco but also across the USA and Europe^2^. Innovative prevention measures are needed to reduce syphilis morbidity among MSM, and thus avoid spreading to a larger population. Previous work on sexually transmitted diseases has shown that sexual contact networks can be very useful to tailor mitigation strategies^3^.

The San Francisco Department of Public Health (SFDPH) maintains legally mandated case-based surveillance for syphilis which includes collected sociodemographic, treatment, and contact tracing information on reported and investigated syphilis cases. This surveillance system allows for the description of sexual networks among reported cases that are investigated. Each pair of individuals who have had a sexual encounter at least once in a time span are connected to each other with a link. The number of early syphilis cases in San Francisco has increased steadily from 26.6/100,000 cases in 2007 to 157.1/100,000 cases in 2015. Network theory indicates the importance of core individuals in sustaining sexually transmitted infections (STI) epidemics. An algorithm was developed in ref. 4 to identify the sexual contact network in the MSM community of San Francisco from case data routinely collected by the SFDPH to better understand the epidemiology of recent syphilis cases and explore possible new approaches for disease control. A sexual contact network with 2,428 nodes and 2,046 links was created. Within this network, a total of 457 disconnected components were identified. Of these, 78% consisted of only 2 or 3 individuals. Eleven components of 10 or more clients were identified, including a large connected component of 953 individuals. Clients in this largest component were more likely to be HIV-positive (*P* < 0.001 from Chi-square) and to have had more cases of syphilis in the past (*P* < 0.0001 from ANOVA) than clients belonging to smaller connected components^4^.

A partner notification (PN) system is a process in which an infected individual (called an index case) notifies (directly or indirectly) his partners (neighbors in the sexual network) of his infectious state^5^. It is hoped that partners of the index case will then seek evaluation and possible treatment (alert state). Partner notification is considered the cornerstone of sexually transmitted disease control, aiming at controlling transmission by (1) treating exposed partners, (2) preventing reinfection of the index cases, and (3) preventing infection of healthy partners. However, public health professionals face many challenges in partner notification, especially in populations using social media as a primary communication venue, or when most of the partners are anonymous. Partner notification can be performed in person, by phone, by mail, or by online systems (e.g., inSPOT^6^, and can take advantage of the knowledge obtained through network analysis to enhance its efficacy.

To evaluate theoretical mitigation strategy effectiveness, mathematical and simulation models have been successfully used. Biological epidemiology has produced a significant number of deterministic and stochastic models^7–9^. In the search for more accurate models, individual-based epidemic models were proposed, in which the contact network is represented by a graph. Individual-based models were successful in relating critical aspects of the epidemic dynamics to the structural properties of the contact network^10–14^.

Syphilis transmission characteristics and disease evolution are well described by the susceptible-infected-susceptible (SIS) model due to the possibility of re-infection after recovery. In the SIS model, each individual can either be susceptible or infected. Transitions for an individual are determined by the state of the individual and his/her neighbors in the sexual network. For the SIS model, as well as for other models, a threshold phenomenon exists such that the infectious disease spreads and become endemic under some conditions of the parameters. A good estimation of the SIS threshold is proved to be equal to the inverse of the largest eigenvalue of the adjacency matrix of the sexual network under a mean field approximation^15, 16^.

Change in human behavior in response to infectious diseases has been a focal point of the behavioral science community for more than 50 years. Modeling human reactions to the spread of infectious diseases is an extremely important topic in current epidemiology, and has attracted substantial attention^17, 18^. In general, human preventive responses to an epidemic spread can be categorized into the following three types: (1) changes in the system state, (2) changes in system parameters, and (3) changes in the contact topology. A comprehensive review of the existing results that examine the interaction between epidemic spread and human behavior can be found in the survey paper by^19^ and in the book Manfredi & D’Onofrio^20^. To include human reactions, Sahneh *et al*. have proposed the susceptible-alert-infected-susceptible (SAIS) model^21^, an extension of the individual-based SIS model where we add a new compartment (alert) to take into account the change in the behavior of susceptible individuals. Juher *et al*. have studied further this model showing the critical role of the absence of awareness decay^22^. Finally, the impact of an information dissemination network on the spreading of the alertness in the SAIS model is studied in Sahneh *et al*.^23^.

The goal of this paper is to evaluate new notification interventions for the San Francisco syphilis epidemic exploiting the identified MSM sexual network, interventions that can be effective and simultaneously require limited additional costs.

To this end, we adapt the SAIS model to simulate the syphilis epidemic in the MSM community of San Francisco. This model is well suited to represent and test multiple notification strategies.

The contributions of this paper are based on the use of a long-term, high-quality, historical syphilis dataset and cutting-edge network science techniques. In particular:

- Our analysis is unique because it has been collected for more than a decade and is focused on the specific community of MSM at San Francisco where the SFDPH has normalized regular screening over the past years.
- We show the effectiveness of new intervention strategies through extensive simulation. In these new strategies, if the index case belongs to a small set of nodes with the highest network centrality –specifically eigenvector centrality–, an alert message of increased risk of infection is sent to the community, inviting people to increase infection prevention. With this strategy, the infection prevalence can be reduced up to 1/3 when the infection status of only 3% of the total individuals is shared.

## Data and Network Description

In the following three subsections we describe the data used to create the sexual contact network and its characteristics, and we introduce the concept of notification network, an additional layer used to inform community members.

### Data Collection and Formats

The San Francisco Department of Health (SFDPH) routinely investigates reported suspected or confirmed cases of syphilis. In order to maintain the confidentiality of the patient, standard partner services approaches focus on the ego-centric network (who are the sex partners of the index). The named partner does not need to name the index back to be considered a sexual connection. At the Department of Public Health of San Francisco, trained field staff members interview index patients, whose infection status is confirmed by lab tests, provide assurance of appropriate treatment, and collect elicitation of sexual partners by self-reporting. Data are collected in standardized formats and include sociodemographic, substance use, sexual behaviors, and HIV serostatus information and consist of a mix of self-reporting and test-based information. Prior reports of early syphilis to San Francisco STD Prevention and Control are used to determine repeat syphilis cases, while HIV status is based on index self-identification as well as confirmed diagnoses reported to the SFDPH. Residential addresses of patients are geocoded and assigned to locally defined neighborhood boundary files using MapMarker (Piney Bowes, Troy, NY) and SAS v9.3 (SAS Institute, Cary, NC). Index patients residing in census tracts that correspond to the Castro neighborhood, where large numbers of gay-identified MSM reside, are coded as Castro residents. Any partners listed with an address outside San Francisco are considered out of jurisdiction. Numbers and types of sexual behavior are assessed in the *critical period* (3 months before the onset of primary symptoms for primary cases and six months before the onset of secondary symptoms for secondary cases) by standard protocols^1^.

The temporal network data is represented by records with 8 fields describing the interacting individuals (via anonymized ID codes), the interval and frequency with which partners were sexually active, the date at which the case of the patient was open, and the critical period for the STD patients over which at least one contact with the partner was made. Below in Table 1, an example of the format for a data record that corresponds to a node pair is shown. Dates are reported as month/day/year.

**Table 1:** Example of the data record format corresponding to a node pair.

Client ID	Partner ID	First Experience	Frequency	Last Experience	Open Date	Critical Period Start	Critical Period End
478214	482624	6/1/2009	STEADY	7/5/2014	7/8/2014	6/26/2013	6/26/2014

Due to the incompleteness of many reported contact intervals–the intervals over which the patients report being sexually active with a partner –, we have decided to use the critical period (defined as the time interval where syphilis exposure or transmission likely occurred) as a surrogate for the contact period. The critical period (CP) is three months for primary syphilis, six months for secondary syphilis, and twelve months for early latent syphilis. The CP is a standard metric in syphilis interviews. Since the frequency was also reported in a non-quantitative manner we decided to restrict our analysis to the following four fields summarized in Table 2.

**Table 2:** The four fields of the data record considered in our analysis.

Client ID	Partner ID	Critical Period Start	Critical Period End
478214	482624	6/26/2013	6/26/2014

Considering the four fields for 2428 individuals, we can build a *contact network* with links among nodes existing during the critical time periods defined by the data and expressed in days.

## Sexual Contact Network

### Contact duration distribution

**Figure 1:**
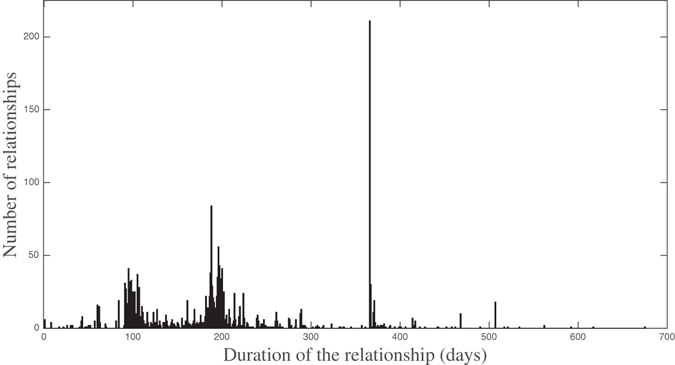
Frequency distribution for the duration of the relationships of the 2428 nodes in the temporal network.

One important issue for understanding the temporal networks characteristics is to study the distribution of the duration of the relationship. Figure 1 shows the frequency of the critical interval durations for all node pairs in the temporal network.

From Fig. 1, it is clearly seen that the distribution of the critical periods has peaks around the standard durations—90 days, 180 days, and 360 days approximately—but also has other durations, even larger than 12 months. This is due to specific circumstances for each index case. For example, the critical period ends whenever patients receive treatment: they could not infect anyone after this time, so we do not need any names of partners. Additionally, the beginning of the CP can be defined as the last date a patient tested negative for syphilis, since he must have been infected some time afterwards. So, if a secondary case had a nonreactive RPR test (standing for rapid plasma reagin, a blood test for syphilis that looks for an antibody that is present in the bloodstream when a patient has syphilis) five months ago, his CP will only be five months long. CP intervals of length greater than 12 months are due to repeated CPs for the same pair of individuals. When we determine that two individuals shared two or more successive critical periods, we assume that the critical period has length equal to the sum of the successive critical periods, obtaining an overall CP longer than 12 months. In particular, there are 158 pairs that share 2 or 3 critical periods.

**Figure 2:**
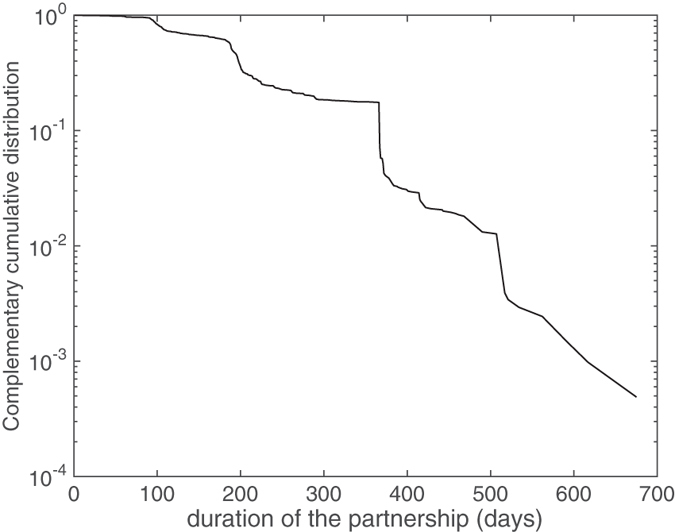
Complementary cumulative distribution of the duration of the partnerships. The roughly linear tail of the distribution indicates an exponential distribution of the highest durations.

The complementary cumulative distribution, *F*(*x*) = *P*(*X* > *x*), of partnership duration is shown in Fig. 2 on a semi-logarithmic plot. For large values of the duration, the distribution roughly resembles a sampling from an exponential distribution because of its linear character. This type of distribution is in agreement with previous studies on sexual partnerships and sexually transmitted infections^24^, and will be the one we will use to generate randomized versions of the weighted contact network built from the data.

### Largest connected component

An aggregated, undirected, and weighted network is then created from the original temporal one with characteristics reported in Table 3. The complete network includes 457 disconnected components, where 280 components have only two nodes (61.3%), 75 components have three nodes, 56 components have a number of nodes included in the range 5 to 9. Only eleven components include more than 9 nodes, and one largest component includes 953 nodes. In Table 3, some network characteristics for this largest component are reported.

**Table 3:** Characteristics and metrics for the complete network and for its largest component.

	Complete network	Largest component
Number of nodes	2428	953
Number of edges	2046	1011
Density	0.000694	0.002
Average Node Degree	1.68	2.12
Node Degree Variance	6.43	13.27
Clustering coefficient	0.014	0.017
Connected components	457	1

**Figure 3:**
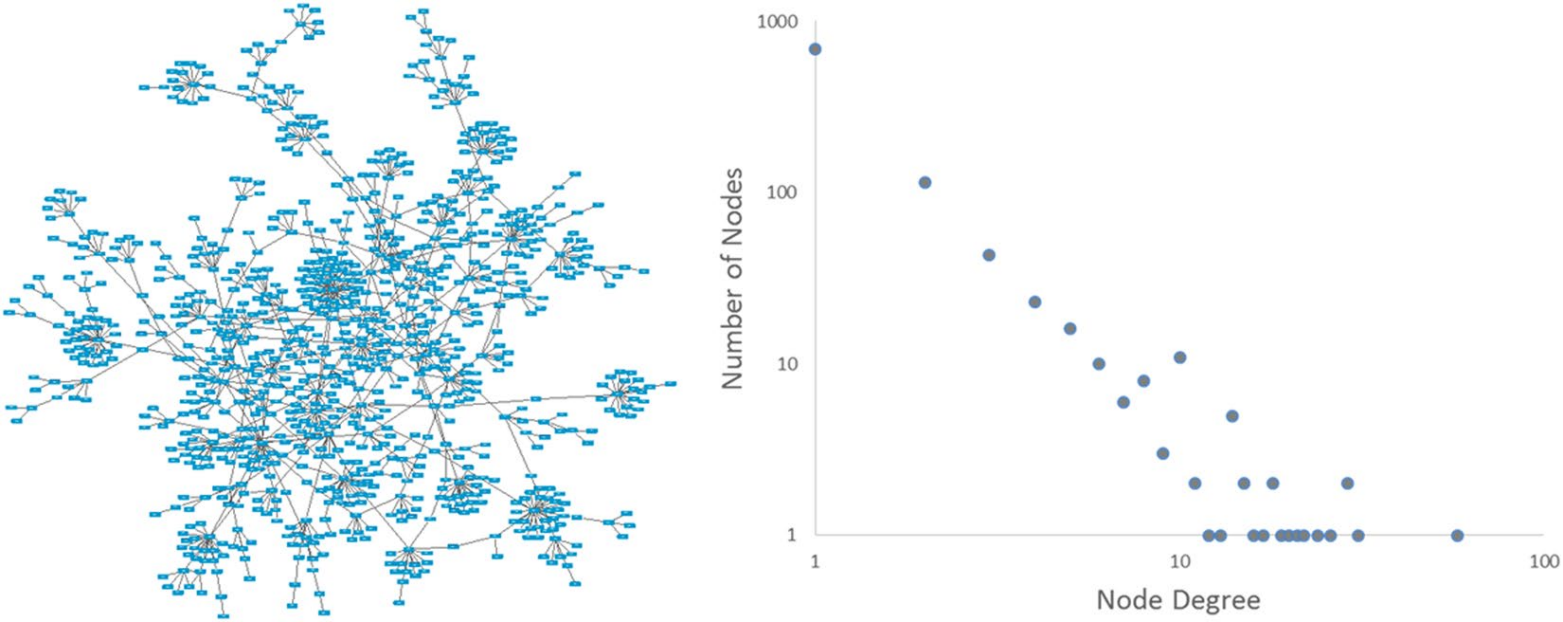
Largest connected component of the sexual contact network (left) and its node degree distribution (right).

In Fig. 3 left panel, the largest connected component of the network is shown. This subnetwork is close to having a tree structure, with a clustering coefficient almost one order of magnitude smaller than the computed clustering coefficient of other sexual networks^25^. We conjecture that this low clustering coefficient could be the result of bias in the data collection, which leads to the presence of several star-like bunches of nodes in the network, that is, nodes with a high number of nearest-neighbors but with a very low number of secondary (next-nearest) neighbors. Additionally, the node degree distribution of the largest component shows a trend similar to a scale-free network in a log-log plot. In fact, hubs are present in this network: one node has degree 58, four nodes have a degree in the range 11 to 13, and eleven nodes have degree 10. On the other side, 808 nodes have degrees one and two. This node degree trend is in line with previous analyses of sexual networks^12^.

### Weighted contact networks

In order to assess the effectiveness of various mitigation strategies based on community alerting, we will carry out stochastic simulations of epidemic spreading until the number of cases reaches a stationary regime. To cover this asymptotic behavior, the contact sequence *S*_0_ = {(*i*, *j*, *t*), *t* = 1, …, *T*} defined on the base of the previous temporal contact records, where *i* and *j* are interacting nodes at time *t*, must be extended for times larger than *T*. There are several ways to do that according to the level of randomization of *S*_0_ (see, for instance^26, 27^).

One of these procedures consists of creating an extended contact sequence for time intervals of the form [*T*, 2*T*], [2*T*, 3*T*], etc., by randomizing the time ordering of the contacts in [0, *T*]. Clearly, this extension of *S*_0_ does not preserve temporal correlations of successive contacts, but the resulting contact sequence has on average the same characteristics as the associated *aggregated weighted network*, a projection along the time axis of the contact sequence *S*_0_ that contains the information on the duration of each contact^26^. The latter is a *static* network that can be used both to carry out stochastic simulations of the epidemic and to compute those centrality measures involved in the definition of alerting strategies. Indeed, although dynamical centralities in temporal networks have been considered elsewhere (see, for instance^28, 29^), they are not especially convenient in the approach we present for defining alerting strategies.

We build the aggregated contact network by assuming that the probability of a contact between two nodes per unit of time is proportional to the duration of the partnership (length of the critical contact period in our data). More precisely, if we consider each record of the contact sequence *S*_0_ as $$[\begin{array}{cccc}i & j & {t}_{ij}^{init} & {t}_{ij}^{fin}\end{array}]$$, the elements (weights) *ω*_*ij*_ of the aggregated adjacency matrix *A* are given by

1 $${\omega }_{ij}=\frac{{t}_{ij}^{fin}-{t}_{ij}^{init}}{{\rm{\Delta }}t}\quad {\rm{with}}\quad \,{\rm{\Delta }}t=\mathop{{\rm{\max }}}\limits_{ij}({t}_{ij}^{fin})-\mathop{{\rm{\min }}}\limits_{ij}({t}_{ij}^{init})$$

that is, Δ*t* is the length of the time period covered by the contact sequence. For the largest connected component, *A* is a 953 × 953 symmetric matrix with 2022 non-zero elements, Δ*t* = 1099 days, and the resulting mean link weight $\bar{\omega }=0.1766$, with a maximum weight of 0.6142 and a minimum weight of 0.0237.

In addition to the aggregated matrix *A*, two other static adjacency matrices will be considered as benchmarks to check the robustness of the results about epidemic spreading with respect to the weight distribution. The first matrix has the same non-zero elements as *A*, but these non-zero elements are here all equal to the mean weight of the contacts (homogeneous adjacency matrix). The second one also has the same non-zero elements as *A* but their values are randomly drawn from an exponential distribution with expected value equal to the mean weight of the contacts. By construction, epidemics on all these three networks will have the same mean transmission rate. We built these two additional static matrices to understand the impact of weight assignments on simulation results. Once we have built these weighted contact networks, the comparison of different alerting strategies will require the computation of centrality measures for ranking nodes according to their influential role in the epidemic dynamics.

### Notification Network

Through the notification network, the infection status of nodes can be notified to their neighbors. In the *standard partner notification* (SPN) strategy, when a client is detected to be infected, all its partners are notified. From this definition of the notification network, it is clear that it has the same topology as the sexual contact network. The only difference consists in the fact that the information network is directed and unweighted, so the SPN network has 953 nodes and 2022 directed links. In general, any notification network will have 953 nodes, but the number of links depends on the specific strategy for notification. In the following, we propose and test multiple new notification strategies.

## The Model

Testing new notification strategies requires a modeling tool which can simultaneously simulate the disease spreading phenomenon and the effect of the partner notification strategy. We propose the use of the Susceptible-Alert-Infected-Susceptible (SAIS) epidemic model defined on a two-layer network where, in addition to the sexual contact layer, a notification layer plays the important role of alerting nodes of the possible risks^23, 30^. In the following, the details of the SAIS model are introduced.

### The SAIS Model with Notification

Syphilis transmission characteristics and disease evolution are well described by the Susceptible-Infected-Susceptible (SIS) model due to the possibility of re-infection after recovery. In the SIS model, each individual can be either susceptible or infected. Transitions for an individual are determined by its state and those of his/her neighbors. An infected node transmits the disease to its neighbors (sexual partners) with a per-contact infection rate *β*, and it is cured with recovery rate *δ*. However, once recovered and healthy, the individual is again susceptible to infection. For the SIS model defined on a network, a threshold *τ*_*c*_ exists such that for *β*/*δ* < *τ*_*c*_ an initial small infection dies out, while for *β*/*δ* < *τ*_*c*_ infection spreads and remains in the population. An approximation of this epidemic threshold is proved to be equal to the inverse of the largest eigenvalue of the adjacency matrix of the sexual network. Based on this relationship, alternative estimates of the epidemic threshold have been proposed for networks with power-law degree distributions^31^.

Modeling human behavioral responses against the spread of infectious diseases in order to improve models’ accuracy is a very complex task and has attracted substantial attention in the last years. In Sahneh *et al*.^21^, the SAIS model was proposed, a *stochastic* epidemic model based on the network-based individual-level SIS model, where an alert compartment is added in order to model the change in the behavior of susceptible individuals. In a first version of the SAIS model, a susceptible node becomes alert with the alerting rate *κ* times the number of infected neighbors in his sexual network. The transition to the alert state is then characterized by three elements: 1) the compartment that induces the transition, which in this case is the infected compartment *I*; the transition rate, in this case *κ*; and the network that defines the neighbors, which in this case is the sexual network *N*_*sexual*_: (*I*, *κ*, *N*_*sexual*_). An alert individual can become infected in a process similar to a susceptible one, but with a smaller infection rate *β*_*a*_ where 0 < *β*_*a*_ < *β* due to the adoption of preventive behaviors.

**Figure 4:**
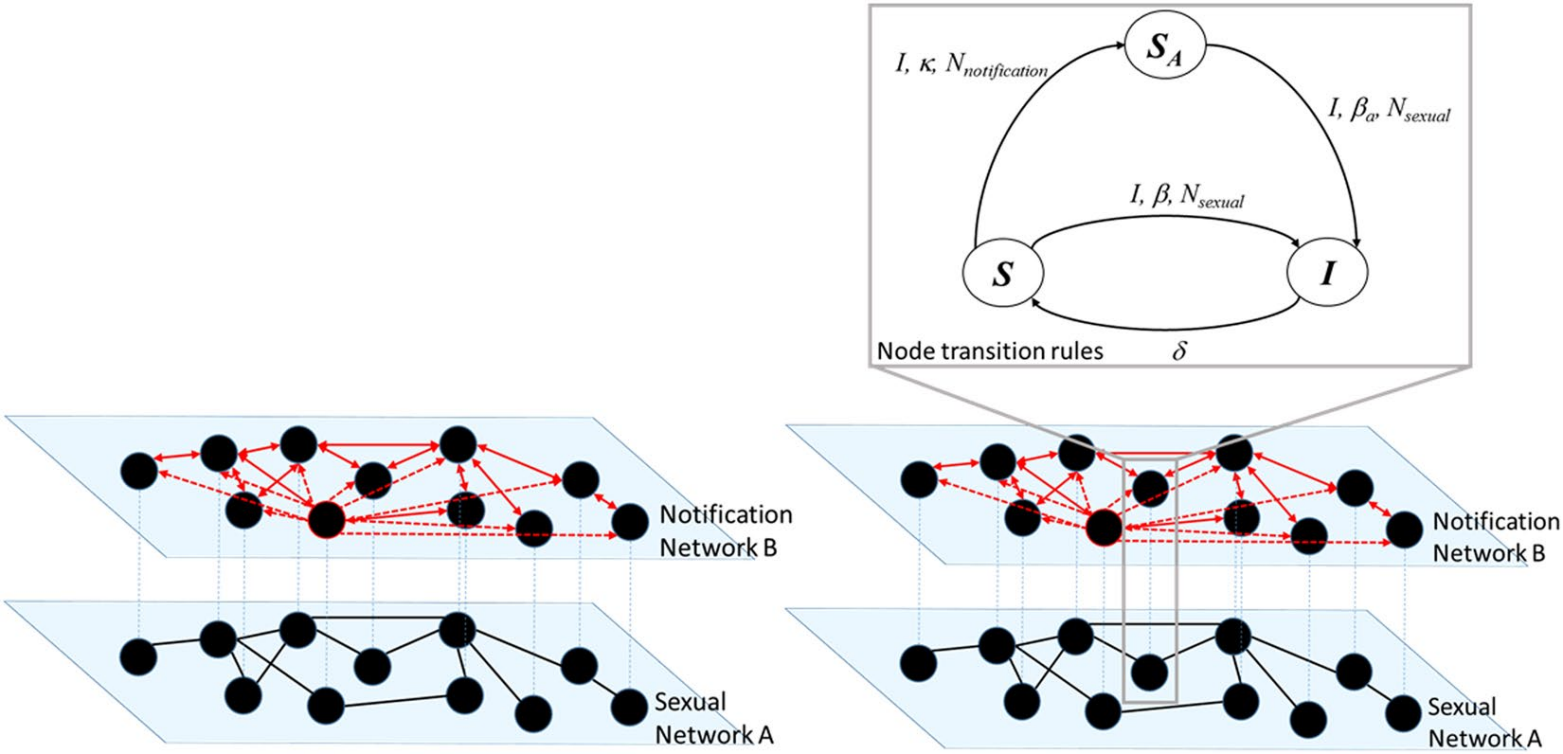
Left: The multi-layer architecture of interactions between individuals. Right: transition diagram for each node in the network among the three states: susceptible (s), alert (s_a_), and infected (i), when the transition to the alert state can be induced by infected neighbors in the notification network.

An extension of the SAIS model incorporates information dissemination social networks in addition to the contact network^23, 30^. Figure 4, left panel, illustrates an example of the two-layer network concept where two types of links, sexual and notification, connect the same set of nodes. In the related *two-layer SAIS model*, individuals can receive infections from infected neighbors in the physical, *sexual network* (black links), and receive notification of current infections through a *notification network* (red links). In the notification network, each node has a (notification) link to all individuals notified of its infectious status. If only physical partners are notified about the infection status of a given node, then the notification network is identical to the sexual network, and there is no difference between the SAIS and the two-layer SAIS model. However, when a susceptible individual might go to the alert state not only if notified of infected partners but also if notified of other core individuals being infected, the two-layer SAIS model shows better disease-control properties than the single SAIS.

Specifically, a susceptible node becomes alert with the alerting rate *κ* times the number of infected neighbors in his notification network. Transition to the alert state is then characterized by the triplet (*I*, *κ*, *N*_*notification*_). Figure 4, right panel, shows details of transition rules of the node given its position in the two networks. The detailed description and mathematical equations of the exact continuous time Markov process and the corresponding approximated mean-field ordinary differential equations for the SAIS, and the two-layer SAIS can be found in the modeling framework introduced in refs 23 and 30.

The time evolution of an epidemic is performed by means of continuous-time stochastic simulations on a two-layer network of 953 nodes. At each time step, one of the competing events –infection (either of a susceptible or an alert), recovery, and alerting– will be selected according to the probability that, given the configuration of individual states in the network at that moment, the event will occur in the next infinitesimal time interval. For each event, this probability is proportional to the number of the potential transitions in the network times the corresponding transition rate. This quantity is the so-called *propensity* of this event at that moment. Once the event has been decided, the involved node, or pair of nodes if infection or alerting is selected, is randomly chosen among those who can experience the transition. Finally, the moment when this transition occurs (i.e., the time increment in the simulation) is drawn from an exponential distribution with expected value equal to the inverse of the sum of propensities of the competing events. For more details of this and other related algorithms, see Gillespie^32^.

For each set of parameter values, the epidemic is repeated 350 times up to time t = 250 to obtain the mean number of infected nodes and the 95% confidence intervals (error bars in the figures). At the beginning of each simulation run, 47 nodes are infected at random (about 5% of the total) to preclude stochastic extinctions of the disease in the initial phase of the epidemic.

Table 4 shows the values of the transition rates used in all the simulations of the SAIS model presented in the paper. The selection of the values of model parameters has been performed on the basis of the following considerations. Parameters *β* and *δ* are selected to obtain comparable incidence results between the model and the data at the SFDPH. Parameter *β*_*a*_ can potentially span all values in the range [0, *β*]; we selected *β*_*a*_ equal to half of *β* as an average value. Finally, we selected a small value of *κ* to represent the difficulties in convincing people to adopt preventive behaviors and as a less favorable scenario for our simulations.

**Table 4:** Transition rate values used in all the simulations of the SAIS model.

Parameter	*β*	*β*_*a*_	*κ*	*δ*
(1/day)	0.94	0.47	0.02	0.30

With the parameter values in Table 4, simulations converge in the long term to a non-zero number of infected individuals, representing the number of cases in the current endemic–like state. After 350 simulation runs, the mean number of infected nodes in the empirical network is 148.

The objective of the following analysis is to determine new notification strategies to reduce this mean number of cases in the long term with contained costs.

### Developing Network-Centric Community Alerting Strategies

In this section, we consider notification strategies that mainly have the goal of alerting the community of the infection status of a central node, who is not necessarily a direct partner. We call these notification strategies as *alerting strategies*. The objective of this part of the study is to identify and develop key metrics to guide alerting strategies. While partner notification has the goal of contacting all sexual partners of an index case, alert them, and treat them if they are in turn infected, community alerting has the goal of alerting some nodes in the network in addition to the sexual partners when the index case belongs to a small subset of core nodes in the network. When the alerting strategy notifies and alerts the entire community, the strategy becomes a *community alerting* strategy. With community notification, the identity of the index case will not be revealed, and only a generic alert message will be sent to the community with the purpose of inviting everyone to adopt effective preventing measures and to look for testing and treatment if needed. We assume that a susceptible individual can become alert  when he receives the alert message, thus reducing his infection probability by adopting preventive measures. Mathematical analysis of the SAIS model produces a very simple and interesting theoretical result under this assumption: core individuals can greatly help contain the infection effectively and promptly if the community is alerted when they are infected^23, 30^.

### Community Alerting Strategy

We have defined a *community alerting strategy* as a notification strategy where the community is alerted to an increased risk of reported syphilis and invited to take preventive actions when few *central nodes* are infected. The definition of “central nodes” depends of course on the used centrality measure that, in general, assigns a relative “importance” to each node in the network. In this paper we will consider three different centrality criteria labeled as DC, WDC, EC (standing for Degree, Weighted Degree and Eigenvector centralities) that respectively assign to each node *v*: (DC) the degree of *v*; (WDC) the sum of the weights of all links connected to *v*; (EC) the magnitude of the corresponding component of the principal eigenvector of the weighted adjacency matrix. For a detailed description of measures and metrics in networks, see Chapter 7 in Newman^33^.

The above mentioned community notification can happen in the real world through the use of a cell phone application whose users are the community members. Theoretical results on the approximated mean-field SAIS model with notification show that the most effective community alerting is realized when the community becomes alerted as a consequence of the infection of any of the nodes with the highest centralities. In other words, the notification network will include *C* nodes that—when infected—will allow the community to be notified of high infection risks. In this theoretical scenario, a limited number of core individuals are identified. Therefore, the alerting strategy consists of notifying all community members when one of these *C* central nodes is infected. Under this assumption, we can ask the next questions: What is the *minimum* number of nodes that must be selected to have a *significant* reduction of the disease? How can we select these *C* nodes?

**Figure 5:**
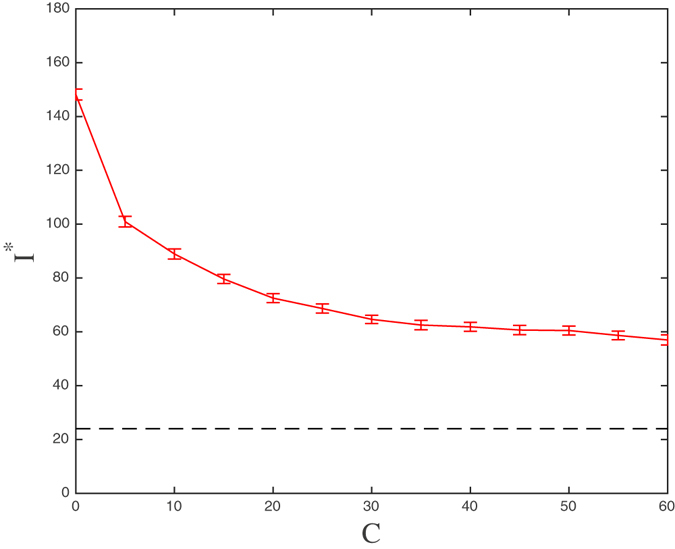
The mean number of infected individuals at equilibrium (and 95% confidence intervals) as a function of the number of selected nodes with the highest degrees. The dotted line shows the number of cases when all nodes are selected (*C* = 953).

To answer the first question, we plot the average number of infectious nodes at the stationary state against the number *C* of central nodes used in the community alerting. Figure 5 shows this relationship for the parameters values in Table 4, and for a selection strategy based on DC, i.e., selecting the *C* nodes with the largest number of connections (links). From the figure, it follows that the reduction of disease prevalence is about 2/3 of the maximum possible for *C* = 30. For values of *C* larger than 30, the reduction in the number of cases is less significant and, moreover, differences between different selection strategies will be progressively less pronounced. In particular in this case, differences between DC and WDC almost disappear. So, to carry out the comparison of alerting strategies, we will consider *C* = 0, 5, 14, and 30. Note that the minimum prevalence will be reached when selecting all the nodes (dotted line in the figure).

An answer to the second question about how we should select the central nodes is given in Table 5. This table summarizes the effectiveness of the strategies of selection of *C* nodes based on the DC, WDC and EC criteria, plus a “blind” strategy *Rand* based simply on selecting *C* nodes at random.

**Table 5:** Mean number of infected individuals in the long run under each selection strategy –Random, Degree Centrality, Weighted Degree Centrality, and Eigenvector Centrality– and the corresponding number of additional links with respect to the standard partner notification (SPN) network for the chosen values of selected nodes *C* = (5, 14, 30).

	*C* = 0	*C* = 5				*C* = 14				*C* = 30			
	SPN	Rand	DC	WDC	EC	Rand	DC	WDC	EC	Rand	DC	WDC	EC
Additional links	0	4754	4587	4622	4739	13312	12980	13013	13285	28491	28015	28056	28483
Number of infected individuals	148	123	100	91	87	105	81	72	64	94	64	63	53

We computed the mean number of infected individuals in the long run under each selection strategy and the corresponding number of additional links with respect to the standard partner notification (SPN) network for the chosen values of *C* (5, 14, 30). The conclusion is that the EC based policy can reduce the average number of infected individuals in the endemic-like state from 148 to 53 when *C* = 30, showing to be the most effective strategy among those tested. Moreover, if we take into account the number of additional links needed to alert the community as a measure of the cost of a strategy, and compare it with the reduction in the disease prevalence, it appears that *C* = 14 could be a better choice than *C* = 30 for the size of the set of central nodes: it still amounts to a significant reduction in the number of cases under the EC (84 cases reduction) and, at the same time, involves less than a half of the links used when *C* = 30.

**Figure 6:**
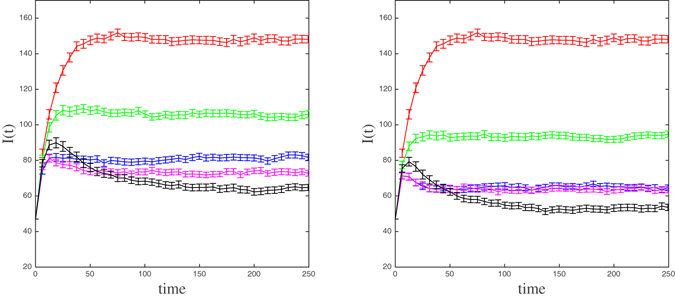
The mean number of infected individuals (and 95% confidence intervals) as a function of time (days) for four selection strategies of 14 (left panel) and 30 (right panel) most central nodes whose infection status it is used to alert the community: SPN (red), Rand (green), DC (blue), WDC (magenta), and EC (black).

In Fig. 6, we report the mean number of infected individuals as a function of time, *I*(*t*), for the same four selection criteria of *C* nodes (*C* = 14 (left) and *C* = 30 (right)). It is interesting to observe that, in both panels, only the black curve representing the number of infected individuals in the EC notification scenario crosses other curves. This means that, for moderately low transmission probabilities as those considered here, a higher initial epidemic growth is expected to occur under the EC selection strategy in comparison to the other two non-random selection strategies. Nevertheless, this non-optimal initial behavior of the epidemic under this strategy is then compensated by a lower number of cases in the long run. In fact, the EC curve decreases reaching the minimum value of infected nodes among the five curves at the steady state regime.

**Figure 7:**
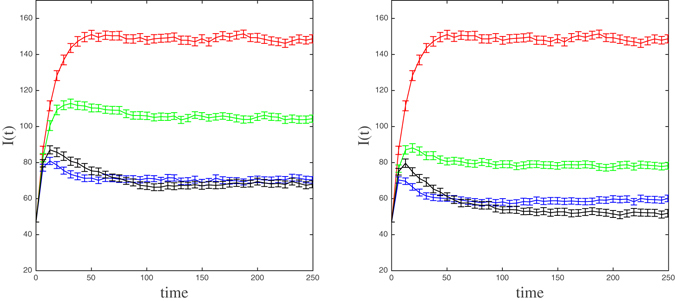
The mean number of infected individuals (and 95% confidence intervals) in the homogeneously weighted contact network as a function of time (days) for three selection strategies of 14 (left panel) and 30 (right panel) most central nodes whose infection status it is used to alert the community: SPN (red), Rand (green), DC (blue), and EC (black).

To assess the consistency of this behavior of *I*(*t*) under different selection strategies, we have simulated the spread of the epidemic on the same network but assigning the mean link weight in the original contact network to all links, namely, $\bar{\omega }=0.1766$. So, the mean transmission rate along a contact is the same on both networks. The results are shown in Fig. 7 for the same number of central nodes as in Fig. 6. Note that, in this case, since the nodes selected by both DC and WDC strategies are the same, the curve for the WDC strategy is redundant.

As before, the initial epidemic growth under the EC strategy is larger than the one under the DC strategy although, at the stationary state, the number of cases is almost the same in both cases for *C* = 14. Differences between levels of the disease prevalence are only noticeable when we select a higher number of nodes (*C* = 30 in the right panel). As expected, the number of infected nodes diminishes with *C* for all the strategies.

**Figure 8:**
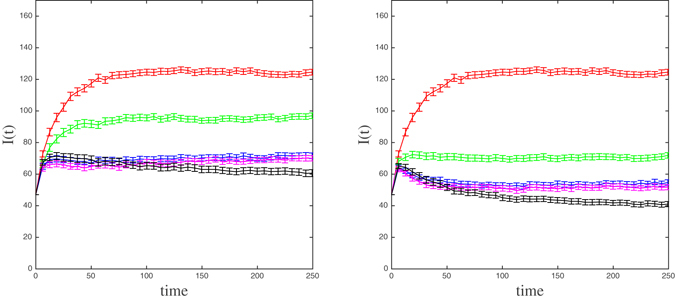
The mean number of infected individuals (and 95% confidence intervals) in the randomly weighted contact network as a function of time (days) for four selection strategies of 14 (left panel) and 30 (right panel) most central nodes whose infection status it is used to alert the community: SPN (red), Rand (green), DC (blue), WDC(magenta), and EC (black).

The consistency of the behavior of *I*(*t*) was also checked by keeping the same topology of the empirical contact network (in particular its degree distribution), but now the weights on the links are randomly assigned according to an exponential distribution with expected value 0.1766, the mean link weight $\bar{\omega }$ (which guarantees the same mean transmission rate along a contact in both networks). Moreover, to preserve the interpretation of a link weight as a contact probability, any generated weight *ω* > 1 is replaced with 1. Note that, for such an exponential distribution, *P* (*ω* > 1) = 0.0035 or, equivalently, only 3.5 occurrences every 1000 generated weights. Figure 8 shows the outcome of the simulations on a particular randomly weighted network. Interestingly, for this and other randomly weighted networks (not shown here), the initial epidemic growth is now pretty much the same under the three selection strategies based on a centrality measure for *C* = 14 and *C* = 30. With respect to the long-term prevalence, EC is again the best of the three strategies for both values of *C* although the amelioration of the prevalence is more noticeable and it happens earlier for *C* = 30.

**Figure 9:**
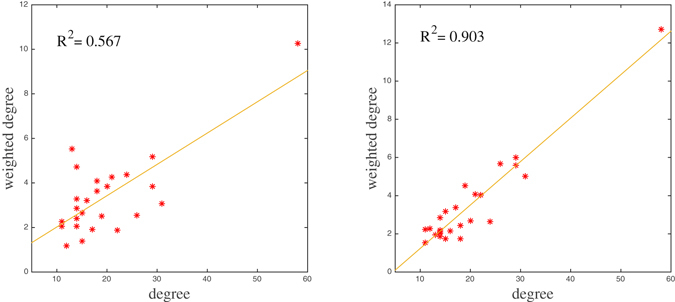
Weighted degrees against degrees of nodes of degree larger than 10 for the empirical contact network (left panel) and the randomly weighted network (right panel) used in Fig. 8.

In contrast to what was observed from the empirical contact network for *C* = 14 (see left panel in Fig. 6), now differences on the evolution of the prevalence under DC and WDC selection strategies are almost not noticeable in Fig. 8 for both *C* = 14 and *C* = 30. Indeed, this is not particularly surprising because there is a high linear correlation between the highest nodal degrees and the corresponding weighted degrees when weights are randomly assigned according to an exponential distribution (in most assignments, the coefficient of determination, *R* ^2^, is larger than 0.75 for nodes of high degree). In Fig. 9 we show this relationship for nodes of degree larger than 10 for both the empirical contact network (*R* ^2^ = 0.567) and the randomly weighted one used in Fig. 7 (*R* ^2^ = 0.903).

Clearly, from this figure it follows that the distribution of link weights among nodes of high degree in the empirical contact network appears to depart from the random exponential distribution. Such a departure implies, first, a low number of common nodes of the two sets of selected nodes and, second, very different centrality values among the non-common nodes. Hence, greater differences in the containment of an epidemic appear when comparing both selection strategies using the empirical data. For instance, for *C* = 10, both rankings have six nodes in common (left panel) whereas, in the example of the random exponential weight distribution in Fig. 8, both rankings share eight nodes (right panel). Moreover, from Fig. 9 it also follows that the nodes with the second and fourth highest weighted degrees in the empirical network are not selected under the DC strategy (non-common nodes).

#### Robustness against missing data

The selection of the most central nodes under each strategy is based on the information provided by the collected data that leads to the construction of the weighted contact network. However, sampling biases and missing data are major problems in empirical studies of hard-to-reach populations^34^. Sampling biases arise because this type of studies is mainly based on chain-referral methods which are examples of nonrandom sampling. For instance, in contact tracing, individuals with many connections are more likely to be nominated by other participants and, moreover, early reported cases have more time for their nominees to be recruited^35^. On the other hand, missing or inaccurate data can occur because of non-disclosure of partners or the existence of “dead-ends” —untraceable contacts— in data collection. In the San Francisco contact network, those sexual partners that are out of jurisdiction (OOJ) are examples of untraceable contacts since they were not included in the subsequent contact tracing.

In what follows, we will assume that there is no bias in the collected data that systematically affects important features of the resulting contact network. In particular, it is assumed that missing nodes are unrelated to the observed data and, hence, they would not significantly change the main structural properties of the sexual network (degree distribution, degree-degree correlation, cyclicality, etc.). Therefore, although missing OOJ nodes can play an important role in disease flare-ups by bringing new cases into the studied area, those properties of the selection strategies depending on the network’s architecture will not be affected by neglecting these nodes in the analysis. For alternative procedures of imputation of missing data see, for instance, Huisman^36^.

On the other hand, the previous results relating the behavior of *I*(*t*) under different distributions of link weights suggest that inaccurate measurement of weights is not a factor that could easily change our findings. So, our major concern has to do with the topology of the contact network. Indeed, the addition of new links increases the cyclicality of the considered network, with possible relevant consequences in the centrality assigned to each node. We claim that the robustness of our strategy under the addition of (missing) links is precisely its main strong point. Recall that the epidemic contention is based on selecting the set *S* of *C* (say, *C* = 14) top-ranked nodes with respect to a given centrality. However, all nodes inside *S* play exactly the same role, so the important question is whether a given individual belongs or not to *S* and in no way its particular position in the ranking. This feature of our contention strategy, combined with the well-established fact that the sexual contact networks are highly heterogeneous, comes to the rescue (see below).

To assess the robustness of the WDC and EC selection strategies against missing links, we have conducted two types of numerical experiments based on the addition of (weighted) links to the original contact network. The idea is to check if (1) the node selection itself is robust against modifications of the set of links, and (2) the containment of the epidemic has the same features as in the original setting when the initial set *S* of *C* central nodes is used. Note that, from a practical point of view, the “true” network is unknown and, so, only the initial set of central nodes can be considered in the experiments.

First, assuming that the set of observed contacts is a random sample from the set of links of the true (and unknown) sexual network, we have randomly added 100, 200, 300, 500, and 1000 links to the original network, which approximately corresponds to a 10%, 20%, 30%, 50% and 100% increase in the number of links, respectively. The random addition of links has been made according to two different attaching mechanisms. In the first one, both ends of the new links are independently attached to nodes that have been selected uniformly at random (avoiding self-loops) from the set of nodes. The second mechanism consists in a semi-preferential attachment of new links by which one end is preferentially attached to nodes of high degree, while the other end is attached to a node selected uniformly at random (again avoiding self-loops). Precisely, following previous observations in growing sexual networks, we have assumed a sublinear preference for the nodal degree *k* with an attaching probability proportional to *k* ^0.7^. This scaling exponent falls within the range of observed exponents in De Blasio *et al*.^37^. So, this second mechanism assumes that under-reported contacts are more related to nodes with higher degrees, although it does not assume correlations between the degrees of the nodes at both ends of a missing link.

Each added link has a weight *ω* that is drawn from an exponential distribution with expected value equal to 0.1766, the mean link weight $\bar{\omega }$ in the original network. As in the previous randomization of link weights, any generated weight *ω* > 1 will be replaced by 1 to preserve its interpretation as a contact probability per unit of time. Since the largest connected component has *N* = 953 nodes and *L* = 1011 links, the initial network has a cyclomatic number *L*–*N*–1 = 57, that can be roughly interpreted as the number of independent cycles in the network. By adding the previous numbers of links, we increase the cyclomatic number to 157, 257, 357, 557 and 1057 independent cycles respectively. We are now in the position to test the impact of “true and unknown networks” with increasing cyclicality on the suggested mitigation strategy.

**Figure 10:**
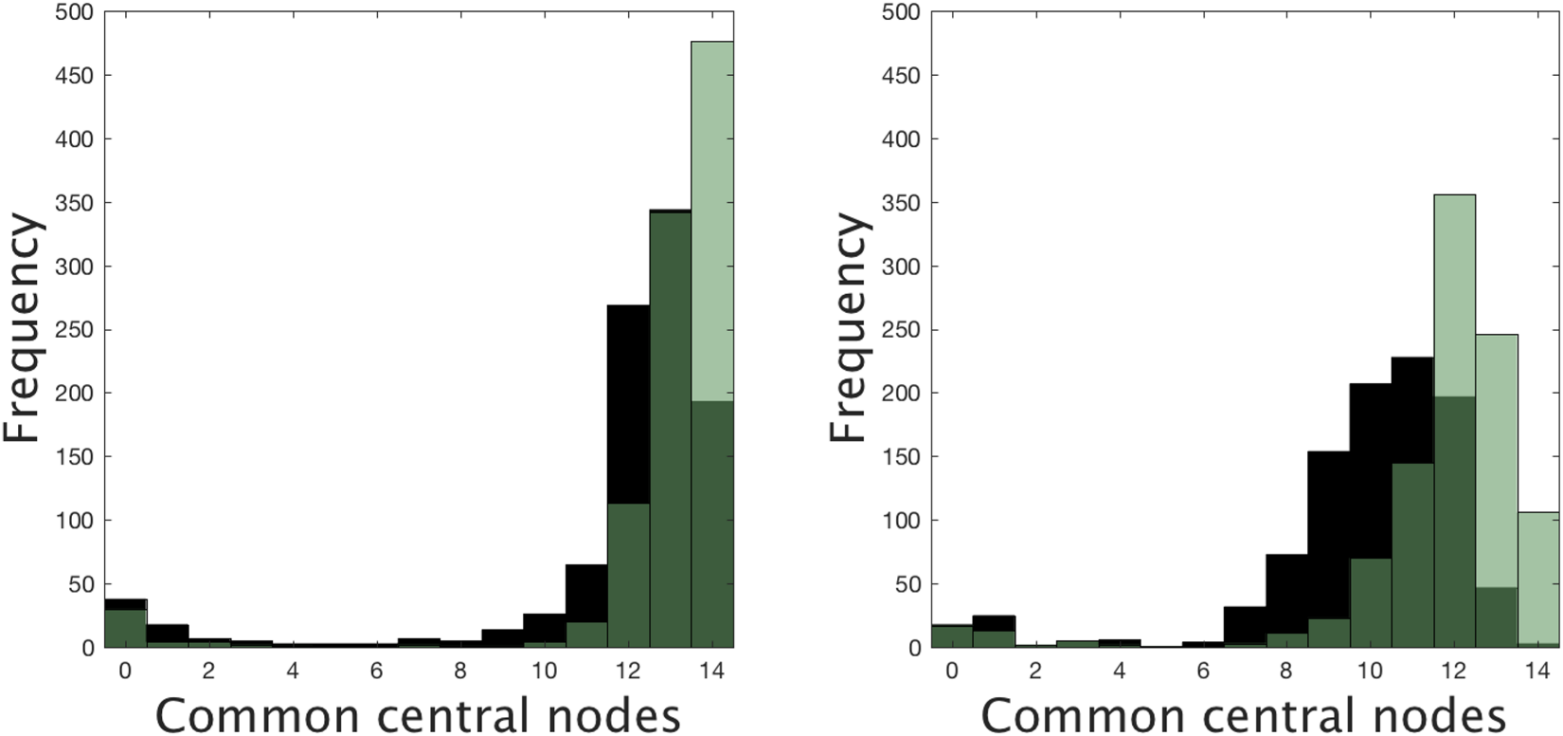
Frequency distribution of the number of shared central nodes under EC selection strategy after adding 500 (green) and 1000 (black) weighted links to the original sexual network under a non-preferential random attachment (left), and under a semi-preferential random attachment (right). Overlapping areas shown in dark green. Experiment repeated 1000 times for each type of attachment and centrality measure, and taking the number of central nodes *C* = 14.

**Figure 11:**
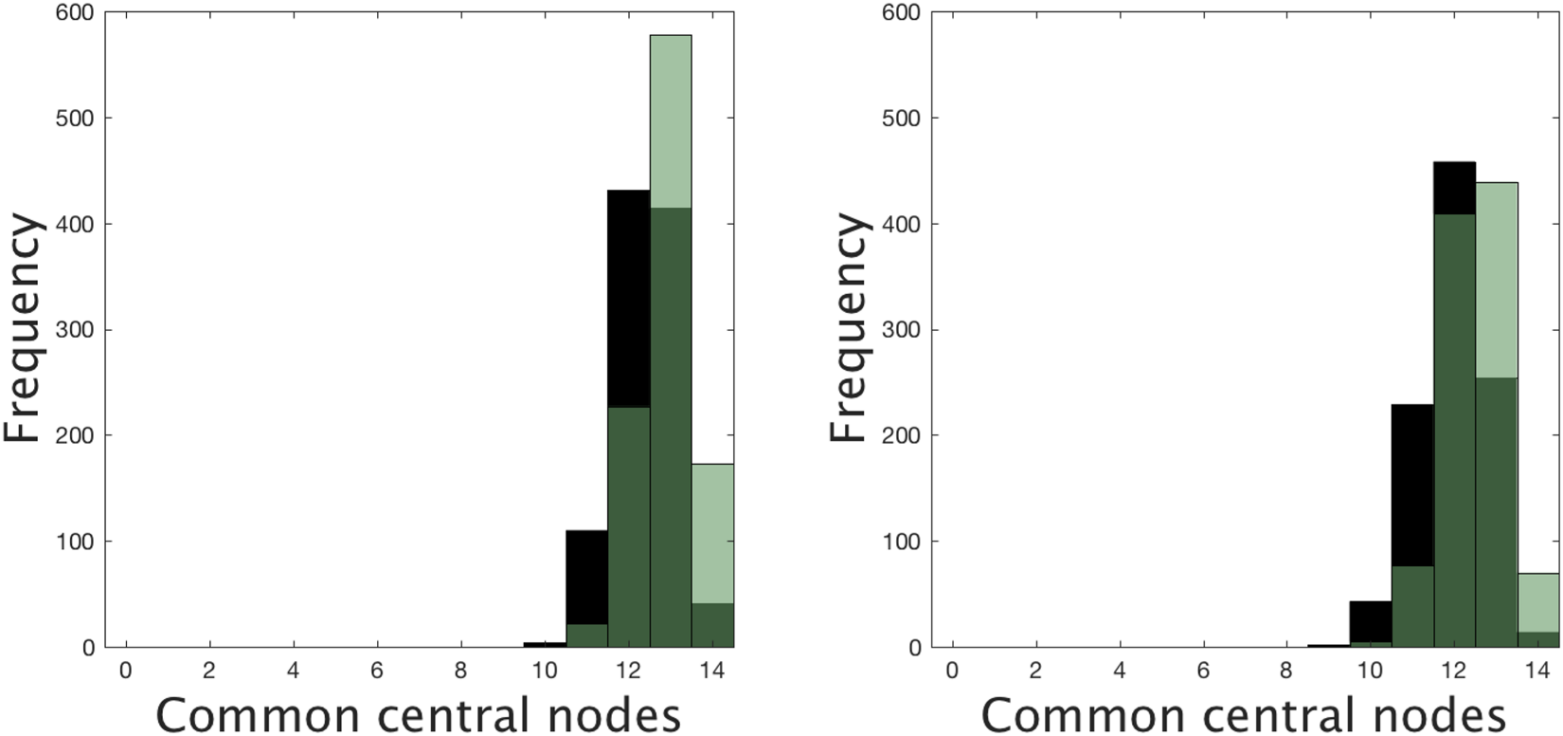
Frequency distribution of the number of shared central nodes under WDC selection strategy after adding 500 (green) and 1000 (black) weighted links to the original sexual network under a non-preferential random attachment (left), and under a semi-preferential random attachment (right). Overlapping areas shown in dark green. Experiment repeated 1000 times for each type of attachment and centrality measure, and taking the number of central nodes *C* = 14.

For each number of added links and for both attaching mechanisms, we have generated 1000 networks and counted how many nodes of the set *S* are among the nodes with the highest *C* = 14 centralities in the new networks, using both EC and WDC selection strategies. Under the EC selection and uniformly random attachment of new links, at least 12 central nodes of *S* are also in the new set of *C* central nodes in more than 93% of the generated networks when we add up to 500 links. When links are added under semi-preferential attachment, the frequency distribution is shifted to the left and it is more left-skewed than before (see Fig. 10). Under the WDC selection, the new set of *C* central nodes always contains at least 11 central nodes of *S* when we add up to 500 links uniformly at random. Under semi-preferential attachment and for the same number of added links, the resulting histogram is slightly shifted to the left but it is still highly concentrated around 12–13 common central nodes even when we add 500 links (see Fig. 11). This high presence of common central nodes for networks with increasing cyclicality shows the robustness of the selection of the central nodes under both strategies.

In contrast to what happens with the WDC strategy, if one analyses the common central nodes selected under the EC strategy, one sees that there are very few times where the number of nodes of *S* in the new set of central nodes is only 0 or 1 (5, 15, 22, 30, and 43 networks when we add 100, 200, 300, 500 and 1000 links, respectively, under the semi-preferential attachment). The reason for such an abrupt change is the distribution of the *C-1* central nodes around the top-ranked one in the original network: all are neighbors of the latter except the node with lowest EC in *S* (which, in turn, has its 14 neighbors in the set of 30 central nodes). Moreover, 9 of these 12 neighbors have degree one. Therefore, when the addition of links leads to an important change in the centrality of the top-ranked node, its neighbors will also be dragged off the top positions in EC and the composition of the new set of central nodes will be very different from that of *S*: 0 or 1 coincidences.

**Figure 12:**
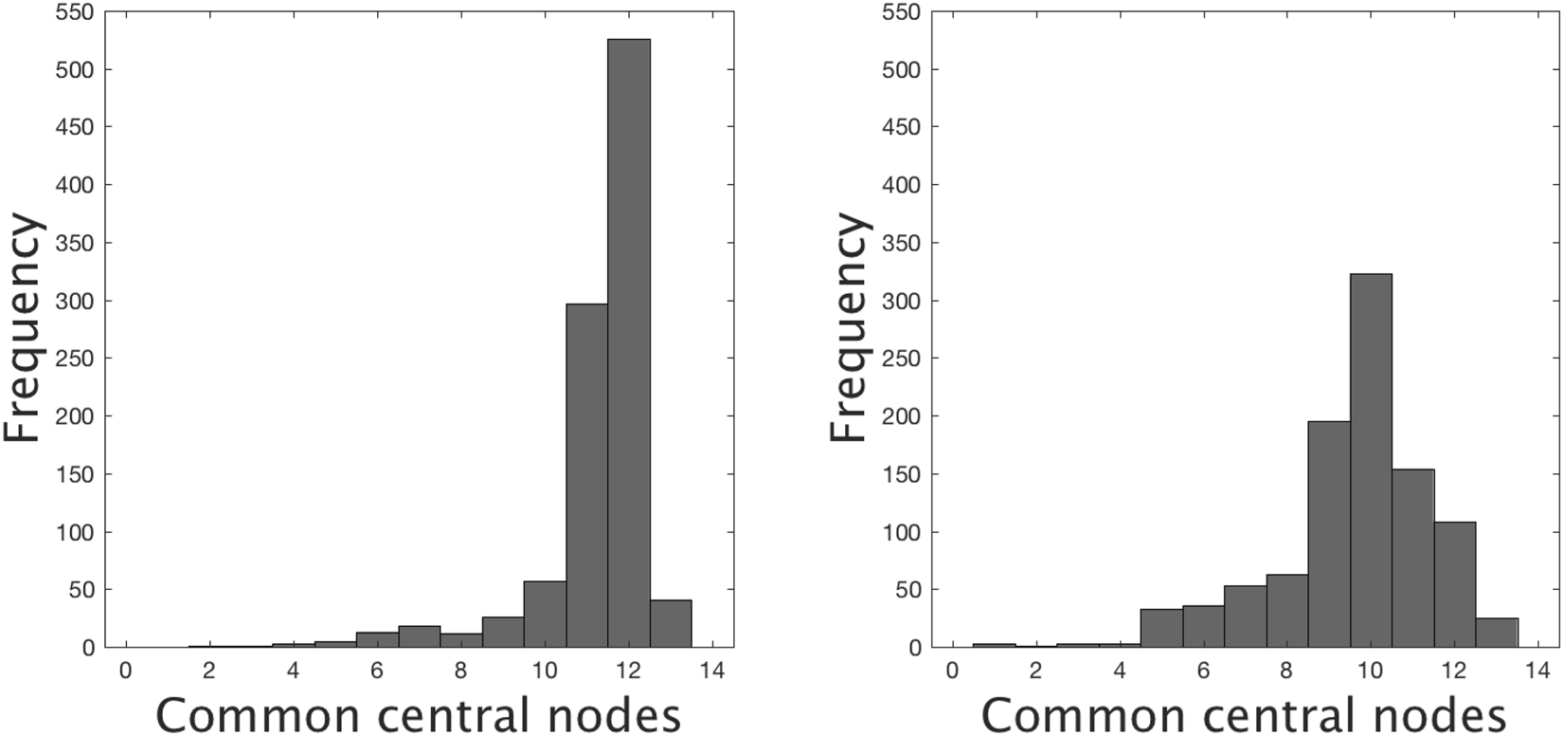
Frequency distribution of the number of shared central nodes under the EC selection strategy after adding 600 weighted links to the same scale-free network under a non-preferential random attachment (left) and under a preferential random attachment (right). Experiment repeated 1000 times for each type of attachment and taking the number of central nodes *C* = 14. For details see main text.

The key point here is that, even though EC is more sensitive than WDC to changes in the number of links, under a random addition of a moderate number of links such a sudden change in the composition of the set of *C* top-ranked nodes is very unlike when the original network is highly heterogeneous. To illustrate this fact, we have generated 1000 networks of *N* = 953 nodes from an initial network with a power-law degree distribution with exponent −3 and mean degree 3.89. For this setting, the configuration model gives initial networks that are connected and close to that of the sexual contact network in Fig. 3 (few highly connected hubs, most nodes with a low degree). For example, adding 600 links (about 30% of links) uniformly at random to a scale-free network yields a unimodal histogram with its maximum around 11–13 common central nodes (the exact location depends on the initial network) and, in most cases, there is no network having a completely different set of central nodes. Under preferential attachment of links to the same instance of network, the resulting histogram is shifted to the left and shows a higher dispersion around a lower maximum (see Fig. 12 for a typical example).

With respect to the containment of the epidemic, simulations were performed on networks sharing different numbers of central nodes with the original set and adding different numbers of weighted links (200, 300, 500, and 1000). It is important to note that, if we want to compare the two selection strategies of central nodes (EC and WDC) using the data of the epidemic in San Francisco, the number of cases without any notification strategy must be independent of the missing links: the network size is always 953 and what we approximately know about the epidemic is its number of cases. However, the addition of new links implies an increase of the mean weighted degree of the network and, if *β* is the same in all the numerical experiments, then the epidemic will have higher prevalence in those networks with a higher fraction of new links. So, for each number of added links, we tune *β* such that, in the long run, all the networks share a similar number of infected nodes when no alert individual is present (*β*_*a*_ = *β*). The reference level is given by the original contact network with *β*_0_ = 0.94 (see Table 4), which leads to an endemic state with 185–190 cases. Suitable values of *β* can then be obtained by imposing that the ratio between the new *β* and *β*_0_ is given by the inverse of the ratio of the link number in the corresponding networks: *β*/*β*_0_ = *L*_0_/*L*. So, the values of *β* we have used in the simulations are 0.785, 0.725, 0.629, and 0.472 when the number of added links are 200, 300, 500, and 1000 respectively. For instance, when 1000 links are added and *β*_*a*_ = *β*, the resulting number of infectious nodes in the long run is about 195 when links are added uniformly at random, and it is about 210 under semi-preferential attachment. In general, the previous relation turns out to be better when links are added uniformly at random because this mechanism introduces less variability in the degree distribution than the semi-preferential one. Once *β* is chosen, we take *β*_*a*_ = *β*/2, as in all the previous simulations.

Simulations up to time t = 250  and for *C* = 14 reveal that under the EC selection strategy, when compared to the WDC selection strategy, there is always a higher initial rise in the number of cases. With respect to the disease prevalence in the long term, EC performs systematically better than WDC only under the uniform random addition of 200 links. At the other extreme, WDC performs systematically better than EC when 1000 links are added with semi-preferential attachment. In the others cases, we observed a tendency of having similar levels of prevalence under both selection strategies and, when there are differences between them, the selection strategy that gives a lower prevalence is not always the same but depends on the generated network. Independently of the number of added links and the selection strategy, the number of infected nodes is slightly higher under the semi-preferential attachment (60–90 cases when 200, 300, and 500 links are added) than under the uniformly random addition of links (45–70 cases). In all settings, the determinant facts are the number of added links and the mechanism of attachment, but not the number of common central nodes between the original network and the new one. In conclusion, also when testing the effectiveness of our proposed mitigation strategies in the worst scenarios, the performance of the EC selection strategy of central node degrades gracefully with an increasing amount of perturbation, showing the robustness property.

#### Responsiveness

**Figure 13:**
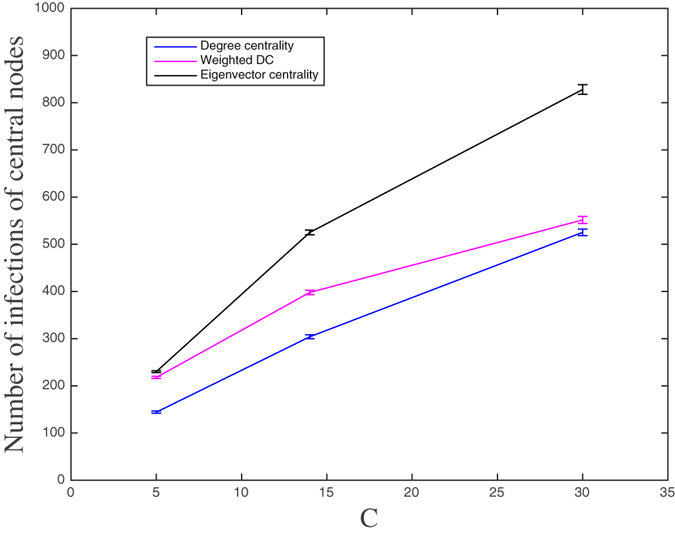
The mean number of infections of the *C* central nodes (and 95% confidence intervals) during an epidemic under the different selection strategies for *C* = 5, 14, and 30. Total time of the simulations: 250 days.

On the other hand, if an alerting message is sent to the community each time one of the central nodes becomes infected, the number of messages received by an individual will be equal to the total number of infections of the central nodes. This could be, in fact, another reason for selecting a low number of central nodes. Otherwise, a decrease in the individual responsiveness might occur because of the saturation caused by a high number of received messages. In Fig. 13, we show this number for the three selection strategies. It is interesting to observe the convergence of the number of infections of the central nodes under DC and WDC strategies when *C* increases from 14 to 30. This is another way to see why DC and WDC selection strategies yield the same level of mean prevalence as *C* increases. On the other hand, the number of infections of the central nodes increases, even more, when *C* increases under EC. This behavior of EC explains why this strategy is more effective for the reduction of the number of endemic cases.

**Figure 14:**
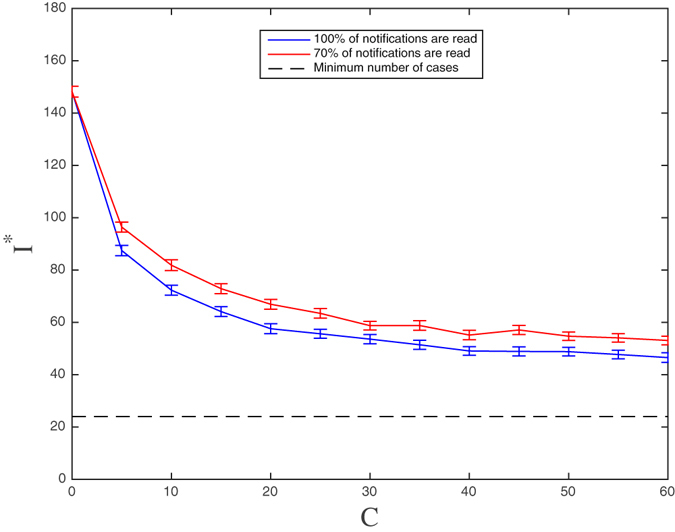
The mean number of infected individuals at equilibrium (and 95% confidence intervals) as a function of the number of selected nodes based on eigenvector centrality (EC) with 100% response (blue line) and 70% response (red line).

Finally, the possibility that an individual receiving an alerting message will completely ignore it needs to be taken into account. To this purpose, we tested the impact on the prevalence of a 30% reduction in responsiveness. We assume here that 70% of the individuals receiving the alert message read it and can decide whether to adopt a preventive behavior or not at a rate *κ*, while the remaining 30% of the individuals who receive the message just ignore it. The lack of response to the alerting message is modelled as the lack of the corresponding communication link. The individuals who miss responding to the alert message are randomly selected. As shown in Fig. 14, a modest increase in the mean number of cases is shown when reducing the number of links by 30% for each value of *C* under the highest eigenvector centrality (EC) strategy. For example, when *C* = 30, the prevalence increases from 53 infected individuals to 59 individuals in the steady state. This can be considered a further robustness feature of the approach.

The selection of the best notification strategy depends on the practical aspects of its implementation. If the notification is performed with a mobile phone app, minimizing the number of links is not a critical goal and strategies like “broadcast the alert message if any of the 30 nodes with the highest eigenvector centrality is infected” seems to be the most promising. However, if saturation in responsiveness can occur as a consequence of the high number of notification messages an individual receives, lower values of *C* could be more suitable, such as *C* = 14.

## Discussion

While the standard partner notification strategy has the goal of testing and alerting the sexual partners of an infected individual, here we extend the notification/alerting component of this strategy to the entire community—community alerting strategy—but limit the alert-triggering events to the infection status of a small number of central individuals. In other words, susceptible individuals will be notified of an increased risk of infection due to the infection status of few central nodes and they will become alerted while still susceptible with a rate proportional to the number of infected central nodes. Critical decisions for community alerting are the number of central nodes selected and the considered centrality measures. The selection of central nodes is performed according to three different centrality measures: degree centrality, weighted-degree centrality, and eigenvector centrality. In contrast to previous studies where the comparison of these centrality measures in an epidemiological context was in terms of the influence of removed (vaccinated) nodes on network topology^38^, we have examined their impact on the evolution of the number of infected nodes *I*(t) on a network where alerted nodes are not removed but can be infected with a reduced rate. To simulate the effectiveness of community alerting strategies we have used a computational model where the rules for the epidemic spread are those given by the transitions between nodal states in a susceptible-alerted-infectious-susceptible model with an alert dissemination network layer. Alerted individuals can be infected at a rate equal to one half of the rate at which susceptible individuals can be infected. Moreover, stochastic simulations are carried out on the largest connected component (953 nodes) of an empirical aggregated sexual contact network of the MSM community in San Francisco.

In all the considered situations and using the empirical MSM contact network, the selection strategy based on the eigenvalue centrality of nodes is the one that leads to the lowest prevalence of the disease in the long run. This strategy takes advantage of the more influential role of nodes high on eigenvector centrality. However, for moderately low transmission probabilities, the initial epidemic growth under DC and WDC alerting strategies is lower than the one under the EC strategy. This seems to indicate that the former strategies are more efficient to contain the epidemic at its early stage. The most vulnerable nodes to contract the disease during an epidemic outbreak are those with the highest degrees which may explain why this occurs for moderately low transmission probabilities. For higher transmission probabilities, however, such differences in vulnerability seem to be not relevant because there is a monotonous ordering of the trajectories of *I*(t), with the EC strategy being always both the one with the lowest initial epidemic growth and the one with the lowest prevalence at the long term. The same behavior of the trajectories of *I*(*t*) is observed when all the link weights in the network are replaced by their mean weight. However, when simulations are performed on a randomized version of the empirical contact network where link weights are assigned according to a random exponential distribution with the same mean weight (while keeping the same network architecture), the three selection strategies lead to very similar initial epidemic growths for the same moderately low transmission probabilities used in the empirical network. This seems to suggest the importance, at this early stage of an epidemic, not only of the degree distribution but also of the pattern of link weights in contact networks. On the other hand, the number of nodes whose state is used to alert the community needs not to be high in order to have a significant impact on the endemic number of cases in the community. With the parameters values used in this study, selecting about 1.5% of the nodes with the highest EC is enough to reduce more than 50% of the prevalence of the disease.

We report that the average degree of the largest connected component is very close to 2 indicating its dendritic structure (a tree graph with the same number of nodes would have a mean degree of 1.997). This feature, along with its very low number of cyclic structures (only nine 2-core groups are detected using the clustering algorithm MCODE –a Cytoscape plugin^39^, and the highly fragmented complete network, has also been observed in other sexual networks^40^. Indeed, it has been claimed elsewhere that this architecture of sexual networks indicates a low level or declining endemic transmission of the infection, in contrast to densely connected structures that are empirically associated with an intense STI outbreak because of the higher probability of STI transmission^40, 41, 42^.

Clearly, these results are obtained under some assumptions the most critical of which are: (1) susceptible individuals become alert with a rate proportional to the number of alert messages received, (2) there is no systematic bias in the collected data and, hence, the set of observed contacts is supposed to be a random sample from the set of links of the true (and unknown) sexual network, and (3) the selected parameters are appropriate. Future work will relax these assumptions, by considering uncertainty in the network topology and weights, exploring behavioral implications of receiving alerting messages and adapting the model consequently, and by fine tuning the model parameters with available incidence data.

In particular, the interplay between awareness decay over time and disease prevalence as well as the determining factors of the former are issues that must be considered for a full understanding of the impact of community alerting strategies. For instance, several public health agencies have reported the re-emergence of some STIs since the mid-1990s, mainly among MSM and in high-income countries, just after the decrease in condom use that followed the introduction of the antiretroviral therapy for HIV in 1996 and the increased use of other non-condom HIV risk-reduction strategies^43^. On the contrary, the usage of face masks as a protection against respiratory infections is still fairly common in several Asian countries since the 2003 SARS epidemic. These two examples show that behavioral responses and their duration may depend both on the culture of the affected population and on the type of disease. The simplest introduction of awareness decay in the context of an SAIS model is to consider a constant decay rate^22^. However, this assumption implies that the average time a behavioral response lasts in an alert individual is equal to the inverse of this rate and, hence, its duration does not depend on the prevalence of the disease in the community.

From the point of view of the implementation of an alerting strategy, it is interesting to take into account that individuals can have a lower propensity to adopt a protective behavior after receiving several alerting messages. Communication technologies (text messaging, Internet PN facilities, phone apps, etc.) provide an instrument for notifying individuals exposed to STIs through web-mediated communities (dating websites, prostitution) and who may not be traceable by other means^5, 44^. However, these technologies also have the potential to end up saturating individuals with an excessive number of alerting messages. A possible way to deal with this progressive saturation could be to structure the population of alerted individuals by the number of messages they receive which, in turn, will depend on the disease prevalence.

In summary, in spite of all possible improvements to the model and its calibration, this paper offers a first look at a novel mitigation intervention in the form of a community alerting strategy that has the potential to be *effective* in reducing the number of cases as shown by the simulations, *cost efficient* since messages to the community can be sent with a cell phone application as anonymous messages concerning increased infection risk, and *practically implementable* since it only requires the knowledge of the infection status of few individuals.
